# Ecosystem services and biodiversity of agricultural systems at the landscape scale

**DOI:** 10.1007/s10661-021-08857-x

**Published:** 2021-05-14

**Authors:** Sonoko D. Bellingrath-Kimura, Benjamin Burkhard, Brendan Fisher, Bettina Matzdorf

**Affiliations:** 1 Leibniz Centre for Agricultural Landscape Research (ZALF), Eberswalder Str. 84, 15374 Müncheberg, Germany; 2 Faculty of Life Science, Humboldt University Berlin, Albrecht-Thaer-Weg 5, 14195 Berlin, Germany; 3 Institute of Physical Geography and Landscape Ecology, Leibniz University Hannover, Schneiderberg 50, 30167 Hannover, Germany; 4 Environmental Program, Gund Institute, Rubenstein School of Environmental and Natural Resources, University of Vermont, 81 Carrigan Drive, Burlington, VT 05405 USA; 5 Institute of Environmental Planning, Leibniz University of Hanover, Herrenhäuser Str. 2, 30419 Hanover, Germany

Agricultural systems all over the world are key for supplying vital goods such as food, fibre and energy from biomass. These agricultural systems form the foundation of human material well-being. However, increasing intensification, monocultures and overexploitation have often led to the serious degradation of the ecosystems upon which agricultural systems are embedded. Therefore, the functioning of these systems and their constituent biodiversity are at risk (Beckmann et al. 2019). Designing more sustainable agricultural systems requires continued research on land use strategies and management that focuses not only on the provision of agricultural commodities but also on the supply of non-agricultural ecosystem services, the conservation of biodiversity and the stable conditions needed for ecosystem functioning (Tilman et al. 2002, Swinton et al. 2007). Agricultural land uses are driven by anthropogenic and natural factors and show site-specific advantages and disadvantages (Power 2010). Agricultural activities have the potential to enhance the provision of specific kinds of ecosystem services and biodiversity, while mismanagement often leads to degraded services, declines in biodiversity and degraded ecosystem conditions (Huang et al. 2015).

Many of the fundamental processes and interactions affecting the long-term sustainability of such systems happen at the landscape scale. For example, pollination services provided by wild bees to agricultural crops increase with the amount of natural area in the farming landscape (Nicholson et al. 2017). As such, at the landscape scale, agro-ecosystems are not only suppliers of benefit for human well-being but also users of various ecosystem services, such as water and nutrient regulation, soil erosion control and pollination (Jones et al. 2016). Understanding this landscape scale is vital to our understanding of specific kinds of ecosystem interactions and, therefore, any potential land use conflicts caused by the divergent needs and preferences of stakeholders across a landscape.

The same management can have different consequences according to the site-specific conditions. However, there is a large mismatch among the spatiotemporal scales where land use decisions are made and where and when the impacts appear (Pelosi et al. 2010). While most of the decisions for management choices are conducted at the plot or field scale, the impacts of these decisions often emerge at the scale of the landscape or larger. Decisions on the use of land are sometimes made by land owners that are located far from the field. Their decisions are often based only on the global prices for demanded commodities such as food, fibre and feed and not on the locally provided ecosystem services and biodiversity. Such mismatches of scales and stakeholders are, however, rarely considered in research and practice. New management approaches are needed to consider all the effects at different scales.

In this Special Issue*,* we address this emergent issue, particularly addressing the following fundamental questions: (a) what kind of land use trade-offs and synergies among agricultural production, ecosystem services and biodiversity can be identified at the landscape scale; and (b) how can the influence of agricultural land use on ecosystem services and biodiversity be monitored and evaluated at the landscape scale? The idea for this Special Issue emerged at the *Landscape 2018 - Frontiers of agricultural landscape research* Conference in Berlin, from where the individual contributions were drawn. The aim of the Conference was to present recent advances in landscape research to promote the development of sustainable agricultural land use and landscape strategies in an interdisciplinary and application-oriented manner.

The research in this Special Issue addresses these landscape-related issues: (a) a general approach to enhance the indication of the supply of provisioning ecosystem services in agricultural landscapes is introduced by Bethwell et al. (2020); (b) aspects of biodiversity are discussed based on approaches from behavioural science in Byerly et al. (2020) and based on the effect on biological pest control (Petit et al. 2020); and (c) ecosystem services relating to soils are presented from aspects including roots (Cebrián-Piquera et al. 2020), soil erosion (Steinhoff-Knopp et al. 2020), soil properties (Cheng et al. 2020) and hydrology (Zikalala et al. 2020). The collection of articles in this Special Issue provides a glimpse of cutting-edge research in agricultural landscape science. It is our hope that such research can aid in the development of more sustainable land use strategies in agricultural landscapes by focusing on the important unit of the landscape.

## References

[CR1] Beckmann M, Gerstner K, Akin-Fajiye M, Ceaușu S, Kambach S, Kinlock NL, Phillips HRP, Verhagen W, Gurevitch J, Klotz S, Newbold T, Verburg PH, Winter M, Seppelt R (2019). (2019): Conventional land-use intensification reduces species richness and increases production: A global meta-analysis. Global Change Biology.

[CR2] Bethwell et al. (2020). Towards an enhanced indication of provisioning ecosystem services in agro-ecosystems. This Special Issue. 10.1007/s10661-020-08816-y PMC8121745 33988773

[CR3] Byerly et al. (2020). Applications of behavioral science to biodiversity management in agricultural landscapes: Conceptual mapping and a California case study. This Special Issue. 10.1007/s10661-020-08815-z 33988766

[CR4] Cebrián-Piquera et al. (2020). Digging into the roots, understanding direct drivers of ecosystem service trade-offs in coastal grasslands via plant functional traits. This Special Issue. 10.1007/s10661-020-08817-x PMC8121717 33988759

[CR5] Cheng et al. (2020). Animal abundance and soil properties affected by long-term organic farming in rice paddies in a typical Japanese yatsuda landscape. This Special Issue. 10.1007/s10661-020-08813-1 33988816

[CR6] Huang J, Tichit M, Poulot M, Darly S, Li S, Petit C, Aubry C (2015). Comparative review of multifunctionality and ecosystem services in sustainable agriculture. Journal of Environmental Management.

[CR7] Jones L, Norton Z, Austin AL, Browne D, Donovan BA, Emmett ZJ, Grabowski DC, Howard JPG, Jones JO, Kenter W, Manley C, Morris DA, Robinson C, Short GM, Siriwardena CJ, Stevens J, Storkey RD, Waters G, Willis F (2016). Stocks and flows of natural and human-derived capital in ecosystem services. Land Use Policy.

[CR8] Nicholson CC, Koh I, Beauchemin A, Richardson LL, Ricketts TH (2017). Farm and landscape factors interact to affect the supply of pollination services. Agriculture, Ecosystems and Environment.

[CR9] Pelosi C, Goulard M, Balent G (2010). The spatial scale mismatch between ecological processes and agricultural management: Do difficulties come from underlying theoretical frameworks?. Agriculture, Ecosystems and Environment.

[CR10] Petit et al. (2020). Landscape-scale approaches for enhancing biological pest control in agricultural systems. This Special Issue. 10.1007/s10661-020-08812-2 33988768

[CR11] Power AG (2010). Ecosystem services and agriculture: tradeoffs and synergies. Philosophical Transactions of the Royal Society B.

[CR12] Steinhoff-Knopp et al. (2020). The impact of soil erosion on soil-related ecosystem services: Development and testing a scenario-based assessment approach. This Special Issue. 10.1007/s10661-020-08814-0 PMC8121738 33988744

[CR13] Swinton SM, Lupi F, Robertson GP, Hamilton SK (2007). Ecosystem services and agriculture: Cultivating agricultural ecosystems for diverse benefits. Ecological Economics.

[CR14] Tilman D, Cassman KG, Matson PA, Naylor R, Polasky S (2002). Agricultural sustainability and intensive production practices. Nature.

[CR15] Zikalala et al. (2020). Hydrological processing of salinity and nitrate in the Salinas Valley agricultural watershed. This Special Issue. 10.1007/s10661-020-08811-3 PMC8121721 33988750

